# Effectiveness of ART optimization on viral load suppression in children and adolescents with HIV in Uganda: A quasi-experimental study

**DOI:** 10.1097/MD.0000000000047862

**Published:** 2026-02-28

**Authors:** Esther M Nasuuna, Lillian Babirye, Joanita Kigozi, Alex Muganzi, Jonathan Izudi

**Affiliations:** a Infectious Diseases Institute, College of Health Sciences, Makerere University, Kampala, Uganda; b Non-communicable Diseases Program, Medical Research Council/Uganda Virus Research Institute and London School of Hygiene and Tropical Medicine Uganda Research Unit, Entebbe, Uganda; c Department of Community Health, Mbarara University of Science and Technology, Mbarara, Uganda; d Directorate of Graduate Training, Research and Innovation, Muni University, Arua, Uganda.

**Keywords:** ART optimization, children and adolescents, dolutegravir, HIV, Uganda, viral load suppression

## Abstract

Uganda implemented the antiretroviral therapy (ART) optimization program in July 2019, based on an eligibility rule. ART optimization targeted individuals with prior viral load (VL) < 1000 copies/mL, while those with VL ≥ 1000 copies/mL continued with non-optimized regimens. We assessed the effectiveness of ART optimization on VL suppression among children and adolescents with HIV (CAWH) in Uganda. We also assessed the compliance of human immunodeficiency virus (HIV) clinics with the eligibility rule and its effect on ART optimization. Therefore, we designed a quasi-experimental study using data from 21 urban and rural HIV clinics. The exposure was ART optimization, defined as the initiation or transition of CAWH on dolutegravir or a protease inhibitor (boosted lopinavir). Children and adolescents with HIV on an optimized ART regimen formed the exposed group, while those on a non-optimized ART regimen comprised the nonexposed group. The primary outcome was VL suppression, defined by VL < 1000 copies/mL after ≥6 months of ART optimization. We assessed the effectiveness of ART optimization on VL suppression using 2-stage least squares instrumental variable regression due to imperfect compliance with the eligibility rule across the clinics. We also established the effectiveness of the eligibility rule on ART optimization for individuals just below and just above the cutoff. Sensitivity analysis was performed using a noncausal approach. We analyzed data from 2999 CAWH aged ≤19 years and found an overall VL suppression of 76.1% (2282/2999). We found that ART optimization showed a trend toward improved VL suppression (risk ratio [RR] 1.81, 95% CI: 0.79–4.14). However, compliance with the rule was only for 2.6% of the participants, and the rule did not improve ART optimization (RR 0.96, 95% CI: 0.88–1.05). Overall, ART optimization, guided by an eligibility rule, did not achieve the target of ≥95% VL suppression among CAWH across the 21 public HIV clinics in Uganda, partly due to low compliance with the rule, although it showed a trend toward improvement. Addressing context-specific biological, behavioral, social, and structural barriers is needed to optimize VL outcomes.

## 1. Introduction

In 2022, an estimated 1.5 million children and adolescents aged ≤14 years had human immunodeficiency virus (HIV) worldwide. Sub-Saharan Africa (SSA) is the hardest-hit region with HIV and accounts for almost 90% of all children and adolescents with HIV (CAWH).^[1]^ In Uganda, CAWH accounts for almost 11% (approximately 15,000) of all people with HIV (PWH).^[2,3]^ In 2020, Joint United Nations Programme on HIV/AIDS released the 95-95-95 ambitious targets aimed at ending the HIV epidemic by 2030.^[4]^ Compared to adults, the third 95 target of ≥95% viral load (VL) suppression among all PWH on antiretroviral therapy (ART) remains distant among CAWH.^[4]^ Viral load suppression is the ultimate goal of ART, and it contributes to preventing heterosexual and vertical HIV transmission, improving the quality of life, and reducing HIV associated mortality and morbidity.^[5]^ In Uganda, the VL suppression rate among CAWH was 42.5% in 2023 compared to 94% among adults with HIV.^[3]^ This suboptimal VL suppression level in CAWH is attributable to a multitude of factors, including unfavorable treatment regimens, weight changes, drug pharmacokinetics associated with increasing age, dependence on caregivers for drug administration, missed clinic visits and ART refills, and psychosocial factors that hinder drug adherence, among others.^[6-9]^

Newer drugs such as dolutegravir (DTG), an integrase strand transfer inhibitor with a high barrier to drug resistance and a favorable therapeutic profile, have been shown in randomized controlled trials to improve VL suppression in PWH.^[10,11]^ Accordingly, in 2019, the World Health Organization recommended DTG-based regimen as the first-line treatment for all PWH.^[12]^ Following this guidance, multiple countries, including Uganda, adopted DTG as the standard first-line regimen through what is known as the ART optimization policy.^[11]^

Uganda introduced the DTG-based policy in July 2019 and implemented it in a phased manner. Transition initially prioritized PWH who were virologically stable, defined as having a VL < 1000 copies/mL, due to limited drug availability.^[7]^ Therefore, VL < 1000 copies/mL on the existing regimen was used as the eligibility rule or threshold for transition to DTG. With sufficient DTG stock levels nationally after July 2019, the policy was expanded to include all PWH irrespective of VL threshold. However, the extent to which the policy was implemented and its effect on VL suppression among CAWH remain unclear.

Therefore, this study evaluated the effectiveness of ART optimization on VL suppression among CAWH across 21 urban and rural HIV clinics in Uganda. Additionally, we assessed the compliance of HIV clinics with the eligibility rule, including the effectiveness of the rule on VL suppression.

## 2. Materials and methods

### 2.1. Description of data and setting

We analyzed routinely collected HIV program data from 21 HIV clinics that offer comprehensive HIV care to over 6000 CAWH. Of the clinics, 6 are urban clinics in Kampala district, and 15 are rural clinics in Western Uganda. The CAWH visited these clinics either monthly or quarterly, and sociodemographic and clinical data were collected and entered into the electronic medical records system. Across the clinics, CAWH aged 0 to 19 years were initiated on ART between January 2002 and January 2022, but the data analyzed were retrieved in June 2022. We retrieved data on residence and categorized as rural versus urban, sex, and age in year then categorized as 0 to 4, 5 to 9, 10 to 14, and 15 to 19 years, point of entry into HIV care, baseline or body weight before ART initiation, baseline ART regimen, ART adherence challenges, ART start date, optimized ART regimen, VL before ART optimization, and ART optimization date. At baseline, participants on protease inhibitor (PI)-based regimens were mainly on abacavir (ABC)/lamivudine (3TC)/lopinavir/ritonavir (LPV/r), ABC/3TC/LPV/r, ABC/3TC/LPVr, zidovudine (AZT)/3TC/LPV/r, and tenofovir disoproxil fumarate (TDF)/3TC/atazanavir/ritonavir (ATV/r), with ABC/3TC/LPV/r being the most common. We also abstracted the first VL data for all individuals following ≥6 months of ART optimization, which we disaggregated as ≤1000 copies/mL or >1000 copies/mL to depict suppressed versus non-suppressed VL, respectively.

### 2.2. Ethical issues

A waiver of informed consent was obtained since this was a retrospective analysis of routinely collected HIV program data. Ethical approval was obtained from the School of Public Health Research Ethics Committee at Makerere University College of Health Sciences (reference number 710), Uganda National Council of Science and Technology (UNCST No. HS553ES), and CDC’s Center for Global Health (reference number: 2019-175).

### 2.3. Study design

We aimed to estimate the causal effect of ART optimization on VL suppression. The most appropriate design for this cause-and-effect relationship would have been a randomized trial. Randomization assigns participants to study arms, ensuring comparability on all measured and unmeasured confounders.^[13]^ However, a randomized trial was not feasible as optimized ART regimens are known from previous trials to improve VL suppression. For programmatic reasons, the Uganda Ministry of Health rolled out ART optimization in July 2019 based on the individuals’ prior VL measurements, but not random assignment. Individuals with prior VL < 1000 copies/mL were prioritized to receive optimized ART regimens, while those with VL ≥ 1000 copies/mL otherwise remained on existing regimen, in accordance with the ART optimization eligibility rule. However, when DTG-based regimens became widely available after July 2019, the eligibility rule was no longer applied, and all individuals with HIV receiving other ART regimens were transitioned to optimized regimens. Therefore, we assessed the effectiveness of ART optimization using a regression discontinuity design (RDD), a quasi-experimental design suitable when intervention assignment is based on an eligibility rule or cutoff.^[14]^ A sharp RDD was initially planned, using the ART optimization eligibility rule (prior VL ≤ 1000 copies/mL) to compare outcomes among individuals just below and just above the cutoff, thereby balancing both measured and unmeasured confounders, and approximating a randomized trial. However, the imperfect application of the rule following adequate DTG stock levels necessitated a fuzzy RDD. The sharp RDD was, thus, employed to assess compliance with the eligibility rule and its effectiveness on ART optimization. The fuzzy RDD estimated the causal effect of ART optimization on VL suppression, treating the eligibility rule as an instrumental variable.

## 3. Variables and measurements

### 3.1. Exposure

The primary exposure was ART optimization, defined as the initiation or transition of CAWH from a non-nucleoside reverse transcription inhibitor based regimen to either an integrase strand transfer inhibitor (DTG) or a protease inhibitor boosted with lopinavir (LPV/r) as the anchor drug.^[7]^

**Figure 1.**
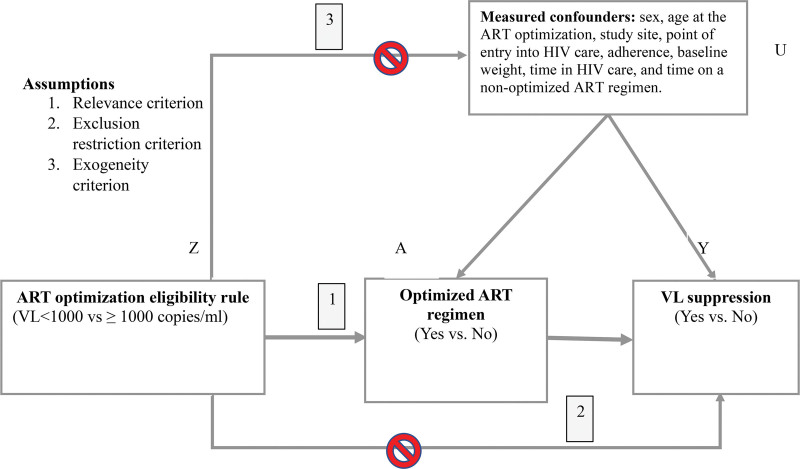
Instrumental variable analysis showing the effect of ART optimization (A) on VL suppression (Y), with ART optimization eligibility rule (Z) as the instrumental variable blocking the confounders (U) from altering the observed association between ART optimization (A) and VL suppression (Y). The key assumptions include the ART optimization eligibility rule, determining the ART optimization (relevance criteria), having no relationship with the confounders (exogeneity criteria), and only affecting VL suppression through its effect on ART optimization (exclusion restriction). The red rings signify blocked causal pathways. ART = antiretroviral therapy, HIV = human immunodeficiency virus, VL = viral load.

Individuals who transitioned to a DTG or LPV/r-based regimen formed the exposed group, while those who remained on existing regimens constituted the nonexposed group. The second exposure was the ART optimization eligibility rule, and it determined whether an individual received an optimized ART regimen or not, as shown in Figure 1.

### 3.2. Outcomes

Viral load suppression following ART optimization was the primary outcome. CAWH with VL < 1000 copies/mL following ≥6 months of ART optimization were considered to have a suppressed VL, while those with a VL ≥ 1000 were considered as having an unsuppressed VL. This was the first VL data following ART optimization.

### 3.3. Covariates

We considered measured covariates, and they included the study site, sex, age at the time of ART optimization, weight before ART initiation, point of entry into HIV care, ART adherence challenges, and the baseline ART regimen. Additional covariates included time in HIV care and time on a non-optimized regimen before ART optimization.

### 3.4. Statistical analysis

Analysis was performed in R Statistical Software and Programming Language (R version 4.2.1; R Foundation for Statistical Computing, Vienna, Austria). We performed a descriptive analysis for the baseline covariates, summarizing categorical data as frequencies and percentages, and numerical data as mean and standard deviation, as the data were normally distributed. We stratified the covariates by ART optimization status and assessed covariate balance using absolute standardized mean differences (SMD) because *P*-values are sample size-dependent.^[15]^ We considered covariates with absolute SMD < 0.1 as suggestive of covariate balance.

To assess compliance with the eligibility rule, we cross-tabulated the eligibility rule (VL < 1000 copies/mL vs VL ≥ 1000 copies/mL) against the ART optimization status, and expressed differences as percentages. Under perfect compliance, all individuals with a VL < 1000 copies/mL would receive the optimized regimen.

We found that the eligibility rule was imperfectly applied, with some eligible individuals not receiving the optimized regimen and some ineligible individuals receiving it. This strengthened the rationale for using a fuzzy RDD^[16]^ and the application of instrumental variable analysis for cause-and-effect analysis.

A valid instrumental variable needs to satisfy 3 conditions as shown in Figure 1: Relevance criterion – a strong association between the instrumental variable (eligibility rule) and the exposure (ART optimization); exclusion restriction criterion – no direct association between the instrumental variable and the outcome (VL suppression) except through its effect on the exposure; and exogeneity criterion – no association between the instrumental variable and measured confounders.^[17,18]^ The eligibility rule was a reasonable instrumental variable because it was correlated with ART optimization (relevance criterion), had no association with VL suppression except through its effect on ART optimization (exclusion restriction), and was not associated with measured confounding factors. Therefore, to establish a causal estimate, the unconfounded association between the instrumental variable and the outcome was used in a 2-stage least squares regression analysis. Here, we fitted a logistic regression model for VL suppression as a function of the baseline covariates and the instrument and computed the probability of ART optimization. We then fitted a modified Poisson regression model for VL suppression as a function of the predicted probabilities and the covariates. Additionally, we conducted subgroup analyses to determine if the effectiveness of ART optimization on VL suppression differed by age groups, namely 0 to 4, 5 to 9, 10 to 14, and 15 to 19 years, and residence (rural vs urban).

To determine the effectiveness of the eligibility rule on ART optimization, we restricted the data within an optimal bandwidth at the cutoff using the Imbens–Kalyanaraman method.^[19]^ This created data for individuals just above and just below the 1000 copies/mL VL cutoff and ensured that both groups were similar on measured covariates. We also computed this causal effect using the modified Poisson regression model. The risk ratio (RR) with a 95% CI was the measure of effectiveness.

### 3.5. Instrumental variable analysis: diagnostics and assumption verification

We assessed the relevance criteria using the *F*-test and considered an *F*-statistic ≥ 10 as indicative of a relevant instrumental variable, as recommended in previous studies.^[16,20-22]^ We assessed the robustness of the causal estimates in a sensitivity analysis by fitting both univariable and multivariable modified Poisson regression analyses, with the latter adjusting for baseline covariates.^[23]^ We reported the sensitivity analysis results as supplementary to the causal estimate.

### 3.6. Reporting

Findings are reported per the improving the reporting quality of nonrandomized evaluations of behavioral and public health interventions: The TREND statement.^[24]^

## 4. Results

### 4.1. Study profile

We retrieved 3756 records for CAWH and excluded 5 of them with no ART optimization status as the data had not been reported, leaving 3751 records. Of the remaining records, we excluded 752 for individuals with no VL data following ART optimization. These individuals had been on ART for <6 months and were therefore not eligible for VL testing, as mandated by national HIV treatment guidelines. Overall, we included 2999 records in the final analysis.

### 4.2. Characteristics of participants and VL suppression

Of the 2999 records for CAWH (Table 1), 96% (2879/2999) were on an optimized ART regimen, 56.5% (1693/2999) were female, and 34.3% (1028/2999) were aged 10 to 14 years. The overall average age was 11.7 ± 4.9 years, and participants on an optimized regimen were significantly older than those on a non-optimized regimen: 11.9 ± 4.8 versus 7.1 ± 5.1, SMD = 0.965, respectively.

**Table 1:** Characteristics of participants and VL suppression.

Variables	Level		Optimized ART regimen		SMD
		All (N = 2999)	No (n = 120)	Yes (n = 2879)	
		Frequency (%)	Frequency (%)	Frequency (%)	
Study site	Rural	1522 (50.8)	101 (84.2)	1421 (49.4)	0.795
	Urban	1477 (49.2)	19 (15.8)	1458 (50.6)	
Sex	Female	1693 (56.5)	72 (60.0)	1621 (56.3)	0.075
	Male	1306 (43.5)	48 (40.0)	1258 (43.7)	
Age categories (yr)	0–4	1014 (33.8)	18 (15.0)	996 (34.6)	1.096
	5–9	658 (21.9)	52 (43.3)	606 (21.0)	
	10–14	1028 (34.3)	10 (8.3)	1018 (35.4)	
	15–19	299 (10.0)	40 (33.3)	259 (9.0)	
	Mean (SD)	11.7 (4.9)	7.1 (5.1)	11.9 (4.8)	0.965
Weight before ART optimization	Mean (SD)	35.55 (16.3)	22.6 (15.3)	36.1 (16.2)	0.858
Point of entry to HIV care	Outpatient department	1894 (63.2)	96 (80.0)	1798 (62.5)	0.442
	Outreach	10 (0.3)	0 (0.0)	10 (0.3)	
	PMTCT clinic	90 (3.0)	0 (0.0)	90 (3.1)	
	Others	1005 (33.5)	24 (20.0)	981 (34.1)	
ART-related adherence challenges	No	2679 (89.3)	103 (85.8)	2576 (89.5)	0.138
	Yes	281 (9.4)	16 (13.3)	265 (9.2)	
	Not reported	39 (1.3)	1 (0.8)	38 (1.3)	
Viral load suppression	No	717 (23.9)	24 (20.0)	693 (24.1)	0.098
	Yes	2282 (76.1)	96 (80.0)	2186 (75.9)	

SMD: absolute standardized mean difference; SMD > 0.1 denotes groups that are different.

ART = antiretroviral therapy, HIV = human immunodeficiency virus, PMTCT = prevention of mother to child transmission of HIV, SD = standard deviation, SMD = standardized mean difference, VL = viral load.

Table 1 shows that systematic differences in ART optimization were observed regarding the study site (SMD = 0.795), age categories (SMD = 1.096), body weight before ART optimization (SMD = 0.858), point of entry into HIV care (SMD = 0.442), and the history of ART-related adherence challenges (SMD = 0.138). The overall VL suppression was 76.1% (2282/2999).

Among individuals on an optimized ART regimen, 75.9% (2186/2879) achieved suppression, compared with 80.0% (96/120) among those on a non-optimized regimen, and this difference was not statistically significant (SMD = 0.098). However, these descriptive figures do not account for selection into ART optimization and confounding, so they should not be interpreted causally.

### 4.3. Effectiveness of ART optimization on VL suppression

In the cause-effect analysis shown in model 1 in Table 2, ART optimization showed a trend toward improved VL suppression (RR 1.81, 95% CI: 0.79–4.14). Sensitivity analysis confirmed these findings, showing no significant association between ART optimization and VL suppression in either the adjusted (aRR 1.00, 95% CI: 0.91–1.09) or unadjusted analyses (RR 0.95, 95% CI: 0.87–1.04). The *F*-statistic was 25.304 (*P* < .001), suggesting that the instrumental variable was relevant for the analysis.

**Table 2:** Effectiveness of ART optimization on VL suppression.

		Model 1 (instrumental variable analysis)	Model 2 (adjusted analysis)	Model 3 (unadjusted analysis)
Variables	Level	RR (95% CI)	aRR (95% CI)	RR (95% CI)
Viral load suppression	No	1	1	1
	Yes	1.81 (0.79–4.14)	1.00 (0.91–1.09)	0.95 (0.87–1.04)

Exponentiated coefficients; 95% CI in brackets; RR: Unadjusted risk ratio. Model 1 is an instrumental variable regression model (a causal model). Model 2 is a multivariable Poisson regression model (a noncausal model). Model 3 is an unadjusted (noncausal model).

aRR = adjusted risk ratio, ART = antiretroviral therapy, CI = confidence intervals, RR= risk ratio, VL = viral load.

### 4.4. Effect of ART optimization on VL suppression in a subgroup analysis

Table 3 shows the subgroup analysis findings. Overall, ART optimization did not show improvement in VL suppression across all age categories. However, the effect differed between rural and urban HIV clinics. In urban clinics, individuals on optimized ART regimens had a large improvement in viral load suppression compared with those on non-optimized regimens (RR 18.0, 95% CI: 1.73–186.95), although the estimate was imprecise. In rural clinics, ART optimization showed a trend toward improving VL suppression among individuals on an optimized ART regimen compared to those on a non-optimized regimen (RR 1.44, 95% CI: 0.97–2.12).

**Table 3:** Effectiveness of ART optimization on VL suppression in a subgroup analysis

			Instrumental variable regression analysis
Age groups (yr)	Variables	Level	RR (95% CI)
0–4	Viral load suppression	No	1
		Yes	37.3 (0.32–4305)
5–9	Viral load suppression	No	1
		Yes	0.57 (0.30–1.07)
10–14	Viral load suppression	No	1
		Yes	0.38 (0.11–1.33)
15–19	Viral load suppression	No	1
		Yes	1.37 (0.66–2.86)
Residence			
Rural	Viral load suppression	No	1
		Yes	1.44 (0.97–2.12)
Urban	Viral load suppression	No	1
		Yes	18.0* (1.73–186.95)

Exponentiated coefficients; 95% CI in brackets.

ART = antiretroviral therapy, CI = confidence intervals, RR= risk ratio, VL = viral load.

* *P* < .05.

### 4.5. Compliance with the eligibility rule and the effectiveness of ART optimization

Of the 2999 participants, 2823 were eligible for ART optimization, while 176 were ineligible based on the eligibility rule. Among those eligible, 2710 (96.0%) received the optimized ART regimen, compared with 169 (96.02%) of those ineligible. Compliance with the eligibility rule was 2.6%. Based on Imbens–Kalyanaraman’s optimal bandwidth of 1030.272 at the cutoff, there were 2823 participants, including 2823 on optimized regimen and 35 on non-optimized regimen. Both categories of participants were similar on all measured confounders. The eligibility rule did not improve ART optimization (RR 0.96, 95% CI: 0.88–1.05).

## 5. Discussion

This study showed that 76.1% of CAWH transitioned on optimized ART regimens achieved VL suppression. Antiretroviral .therapy optimization showed a trend toward improved VL suppression but did not reach statistical significance. Besides, ART optimization showed a significant effect on VL suppression among CAWH who accessed care across urban HIV clinics, but not rural clinics, and across age categories. We found that the compliance of the clinics with the eligibility rule was low and that the eligibility rule did not impact ART optimization.

The suboptimal VL suppression in this study is consistent with findings in several previous studies. A large cohort study involving 3347 children in Mozambique reported a VL suppression rate of 85.8% among those on a DTG-based regimen.^[25]^ A recent study among 2499 CAWH in Nigeria also showed no difference in VL suppression among CAWH on a DTG-based regimen compared to those on other ART regimens.^[26]^ Another study done in Cameroon involving children, adolescents, and adults on a DTG-based regimen similarly found suboptimal VL suppression among CAWH compared to adults, although only 36% of the 225 children were on a DTG-based regimen.^[6]^ A single-center cohort study involving 200 CAWH aged < 19 years found a VL suppression rate of 66.7%.^[27]^ Among CAWH in high-income countries who have been using DTG for a longer period, a narrative review showed that the desired VL suppression rate of ≥95% was also not achieved.^[28,29]^ All these findings are consistent with data that show suboptimal VL suppression among CAWH on optimized ART regimens.

Our findings contrast with those reported in some studies. A large retrospective cohort study conducted across 6 countries in SSA showed a 93.7% VL suppression among 7898 CAWH aged 0 to 19 years on DTG either as a first-line ART regimen or who were ART-experienced.^[30]^ Unlike our study, which included CAWH across different HIV clinics with varying quality of care, depicting real-world settings, the children studied across the 6 countries all attended Centers of Excellence Clinics. Such clinics probably provided better quality HIV care and support compared to public health facilities in our study and in many countries in SSA. A retrospective cohort study of 38 children aged <5 years transitioned to a DTG-based ART regimen in Lesotho found a 94.7% viral suppression rate at 24 months.^[31]^ However, all the studies with contradictory findings had relatively smaller sample sizes compared to our study.

Also, a few of the studies were conducted under controlled conditions, hence the findings do not represent real-world evidence. Achieving a suppressed VL in a real-world or programmatic setting is influenced by not just drug factors but several contextual factors such as the level of immunosuppression at ART initiation, adherence to ART, psychosocial factors like poverty, mental health challenges, and social factors like stigma and discrimination.^[32,33]^ These factors, which hindered VL suppression in prior ART regimens, could exert a similar effect after ART optimization. They may explain the contradictory results in real-world settings across past studies, and also in our study.

The trend towards improved VL suppression following ART optimization aligns with a target trial emulation in South Africa that also found ART optimization tended to improve VL suppression at 12 and 24 months.^[34]^ Similarly, an RDD study reported a small, nonsignificant improvement at 6 months.^[35]^ In Uganda, the analysis of large observational HIV program data for 64,723 CAWH also showed a slight improvement in VL suppression, but the increase remained suboptimal (<95%) despite ART optimization.^[7]^

Dolutegravir showed effectiveness in improving VL suppression under randomized trial conditions. However, in real-world settings, contextual factors shape implementation, including its outcomes such as effectiveness, adoption, acceptability, reach, and sustainability, among others. Contextual factors, therefore, might have to be addressed if evidence-based interventions such as optimized ART regimens are to yield the desired result of ≥95% VL suppression among CAWH. Another important issue to consider is the higher VL threshold (VL < 1000 copies/mL) for ART optimization. The majority of the individuals in the group considered as having a suppressed VL could have had VL near the 1000 copies/mL threshold at the time of ART optimization. Additional exploratory analysis found that CAWH in the optimized ART regimen group had, on average, a 4-fold higher prior VL compared to those in the non-optimized ART regimen group. Therefore, a lower threshold for VL suppression, such as <200 copies/mL, would be more preferable for achieving optimal ART delivery.

The low compliance of HIV clinics with the eligibility rule is explained by programmatic changes or program selection. The rule was initially targeted at individuals with suppressed VL due to insufficient ART stock levels. However, when optimized ART regimens eventually became fully sufficient through the PEPFAR support, the clinics did not comply with the rule, as all individuals received the optimized regimen.

Overall, Uganda successfully implemented the ART optimization policy, and additional measures are now needed to reach optimal VL suppression rates. Therefore, there is a need to strengthen support systems for CAWH and their caregivers to overcome ART-related challenges, such as ART adherence, home-based care, HIV serostatus disclosure, and access to peer support.^[8,33,36]^

## 6. Study strengths and limitations

This study provides evidence on the effectiveness of using an optimized ART regimen on VL suppression among CAWH in a real-world setting. We analyzed a large observational dataset and utilized a rigorous analytic approach that controlled for measured, unmeasured, and unknown confounders. Sensitivity analysis using noncausal approaches showed that the findings are robust. The inclusion of data from both rural and urban HIV clinics makes findings applicable to similar settings in other parts of Uganda and SSA. Limitations include the lack of data on pre-transition drug resistance that could have explained the lack of effectiveness of ART optimization on VL suppression. Also, 5 participants had no documented ART optimization status, as secondary data were analyzed. However, the level of missingness was low, hence unlikely to alter the findings. The study findings should be interpreted in light of these limitations.

## 7. Conclusion

Antiretroviral therapy optimization, guided by an eligibility rule, did not achieve the target of ≥95% VL suppression among CAWH across 21 public HIV clinics in Uganda, partly due to low compliance with the rule, although it showed a trend toward improvement. Addressing context-specific biological, behavioral, social, and structural barriers is needed to optimize VL outcomes.

## Author contributions

**Conceptualization:** Esther M Nasuuna, Joanita Kigozi, Alex Muganzi, Jonathan Izudi.

**Data curation:** Esther M Nasuuna, Lillian Babirye, Joanita Kigozi, Jonathan Izudi.

**Formal analysis:** Lillian Babirye, Jonathan Izudi.

**Investigation:** Lillian Babirye, Joanita Kigozi, Alex Muganzi, Jonathan Izudi.

**Methodology:** Esther M Nasuuna, Lillian Babirye, Joanita Kigozi, Alex Muganzi, Jonathan Izudi.

**Project administration:** Esther M Nasuuna, Joanita Kigozi, Alex Muganzi.

**Resources:** Joanita Kigozi, Alex Muganzi.

**Software:** Lillian Babirye.

**Supervision:** Joanita Kigozi, Alex Muganzi.

**Validation:** Alex Muganzi, Jonathan Izudi.

**Visualization:** Jonathan Izudi.

**Writing** – **original draft:** Esther M Nasuuna, Jonathan Izudi.

**Writing** – **review & editing:** Esther M Nasuuna, Lillian Babirye, Joanita Kigozi, Alex Muganzi, Jonathan Izudi.
